# A Preliminary Quantitative Electron Microscopic Analysis Reveals Reduced Number of Mitochondria in the Infralimbic Cortex of Rats Exposed to Chronic Mild Stress

**DOI:** 10.3389/fnbeh.2022.885849

**Published:** 2022-05-04

**Authors:** Dávid Csabai, Abigél Sebők-Tornai, Ove Wiborg, Boldizsár Czéh

**Affiliations:** ^1^Neurobiology of Stress Research Group, Szentágothai Research Centre, University of Pécs, Pécs, Hungary; ^2^Department of Laboratory Medicine, Medical School, University of Pécs, Pécs, Hungary; ^3^Department of Health Science and Technology, Aalborg University, Aalborg, Denmark

**Keywords:** electron microscope, infralimbic cortex, ultrastructure, quantitative analysis, chronic mild stress (CMS), animal model for depression, medial prefrontal cortex (mPFC), mitochondria

## Abstract

Exposure to severe, uncontrollable and long-lasting stress is a strong risk factor for the development of numerous mental and somatic disorders. Animal studies document that chronic stress can alter neuronal morphology and functioning in limbic brain structures such as the prefrontal cortex. Mitochondria are intracellular powerhouses generating chemical energy for biochemical reactions of the cell. Recent findings document that chronic stress can lead to changes in mitochondrial function and metabolism. Here, we studied putative mitochondrial damage in response to chronic stress in neurons of the medial prefrontal cortex. We performed a systematic quantitative ultrastructural analysis to examine the consequences of 9-weeks of chronic mild stress on mitochondria number and morphology in the infralimbic cortex of adult male rats. In this preliminary study, we analyzed 4,250 electron microscopic images and 67000 mitochondria were counted and examined in the brains of 4 control and 4 stressed rats. We found significantly reduced number of mitochondria in the infralimbic cortex of the stressed animals, but we could not detect any significant alteration in mitochondrial morphology. These data support the concept that prolonged stress can lead to mitochondrial loss. This in turn may result in impaired energy production. Reduced cellular energy may sensitize the neurons to additional injuries and may eventually trigger the development of psychopathologies.

## Introduction

Coping with stress is part of our daily routine, but when stress is severe, long-lasting and uncontrollable then, it becomes a strong risk factor for the development of several somatic or mental disorders. Animal studies provide ample evidence that stress exposure can result in functional and morphological changes of limbic brain areas (McEwen et al., 2015). Pronounced stress-induced changes of neuronal morphology have been documented in the medial prefrontal cortex (mPFC) of rodents such as dendritic debranching, loss of dendritic spines and synaptic contacts (Holmes and Wellman, 2009; Lucassen et al., 2014; Arnsten, 2015; Woo et al., 2021). The infralimbic cortex (IL), which is the most ventral part of the mPFC, has been implicated in several higher order executive functions and it plays a critical role in regulating the chronic stress response (Flak et al., 2012). Neurons of the IL appear to be highly sensitive to environmental stress (Izquierdo et al., 2006; Hinwood et al., 2011; Moench et al., 2015; Czéh et al., 2018).

Mitochondria are intracellular organelles that generate most of the chemical energy needed to power biochemical reactions of the cell. According to recent theories, they also seem to play a key role in the pathological stress response (Morava and Kozicz, 2013; Daniels et al., 2020; Allen et al., 2021). As described by the “mitochondrial allostatic load” theory, chronic stress disrupts the glucocorticoid and glucose homeostasis which in turn alter mitochondrial structure and function (Picard et al., 2014, 2018). Indeed, it has been documented that chronic stress can lead to changes in mitochondrial function and metabolism in the mPFC of mice and that the behavioral profile of the stressed animals correlates with the mitochondrial DNA encoded gene expression in the PFC (Weger et al., 2020). Moreover, it has been proposed that severe, or prolonged stress can result in fragmentation of mitochondria and disruption of the mitochondrial network, which in turn give rise to increased vulnerability of the neurons to further insults and eventually can lead to cell death via apoptosis (Shutt and McBride, 2013; Choi and Han, 2021).

Our aim was to investigate putative mitochondrial damage in response to chronic stress in neurons of the mPFC. We carried out a systematic quantitative ultrastructural analysis to investigate the consequences of prolonged (9-weeks) stress on mitochondria number and morphology in the infralimbic cortex. We subjected rats to the chronic mild stress (CMS) protocol, which is a widely used and thoroughly characterized rodent model for major depressive disorder (Willner, 2005, 2016). It is well documented that rats, in response to CMS, develop behavioral abnormalities that are typical of depressed patients, such as anhedonia (Willner et al., 1992; Wiborg, 2013), cognitive deficits (Henningsen et al., 2009; Martis et al., 2018), or disturbed circadian rhythms (Gorka et al., 1996; Christiansen et al., 2016). In addition to that, a large variety of neuronal changes have been described in the brains of rats subjected to the CMS model (Willner, 2016; Khan et al., 2020). Our hypothesis was that chronic stress exposure will reduce the number and alter the morphology of mitochondria in neurons of the infralimbic cortex.

## Materials and Methods

Eight adult, male Wistar rats (Taconic, Denmark) were used in the present study (*n* = 4 control and *n* = 4 stressed rats). These animals were selected from a much larger cohort. The original experiment involved 335 rats. 260 rats were subjected to the CMS protocol, whereas the remaining 75 rats were kept undisturbed in separate rooms and used later as controls. Stressed animals responded differently to the chronic stress exposure. Many animals developed pronounced anhedonia, a smaller fraction displayed distinct resilience, while most animals displayed intermediate behavioral phenotype. The behavioral phenotyping is described below.

Animals were kept is standard laboratory conditions. Control and stressed rats were singly housed and kept in separate rooms. All experimental procedures were done in accordance with Aarhus University (Aarhus, Denmark) guidelines, Danish and European legislation regarding laboratory animals and approved by Danish National Committee for Ethics in Animal Experimentation (2008/561-447).

The chronic mild stress (CMS) procedures were done according to our standard protocol as described in detail before (Henningsen et al., 2009, 2012; Csabai et al., 2017; Czéh et al., 2018). Briefly, stressed rats were subjected to daily mild microstressors for 9 weeks. The following mild stressors were used: grouping, food or water deprivation, periods of intermittent illumination, stroboscopic light, soiled cage, and cage tilting (45°). During grouping, rats were housed in pairs with different partners, with the individual rat alternately being a resident or an intruder. These microstressors were applied in a predefined schedule over the 9 weeks of CMS and each microstressor lasted for a duration of 10-14 h.

The behavioral phenotyping of the animals was done using the sucrose consumption test (Henningsen et al., 2012; Csabai et al., 2017). This behavioral test enables the detection of anhedonic behavior in response to stress since stress-susceptible animals reduce their sucrose intake indicating a depressive-like anhedonic behavior. Rats were trained to consume a palatable sucrose solution (1.5%) for five weeks before the real experiment started. During this training period, sucrose consumption was measured twice a week in the first two weeks and once per week during the last three weeks. The mean of this last three sucrose intake measurements was specified as the baseline sucrose consumption of the rats. Animals were food and water deprived for 14 h before the test. During the test the rats had free access to a bottle with 1.5% sucrose solution for 1 h. During the 9 weeks of the chronic mild stress period, the sucrose consumption test was performed once per every week. Based on the sucrose intake data, the hedonic state of the animals was evaluated and stressed rats were finally further divided into stress-susceptible rats (anhedonic animals) and stress-resilient rats (Henningsen et al., 2012). Anhedonic animals are the ones that reduce their sucrose solution intake by more than 30% in response to the stress exposure. Stress resilient animals are the ones which do not decrease their sucrose intake during the stress procedures. Data from these animals have been published elsewhere (Csabai et al., 2017, 2018; Varga et al., 2017; Czéh et al., 2018).

**FIGURE 1:**
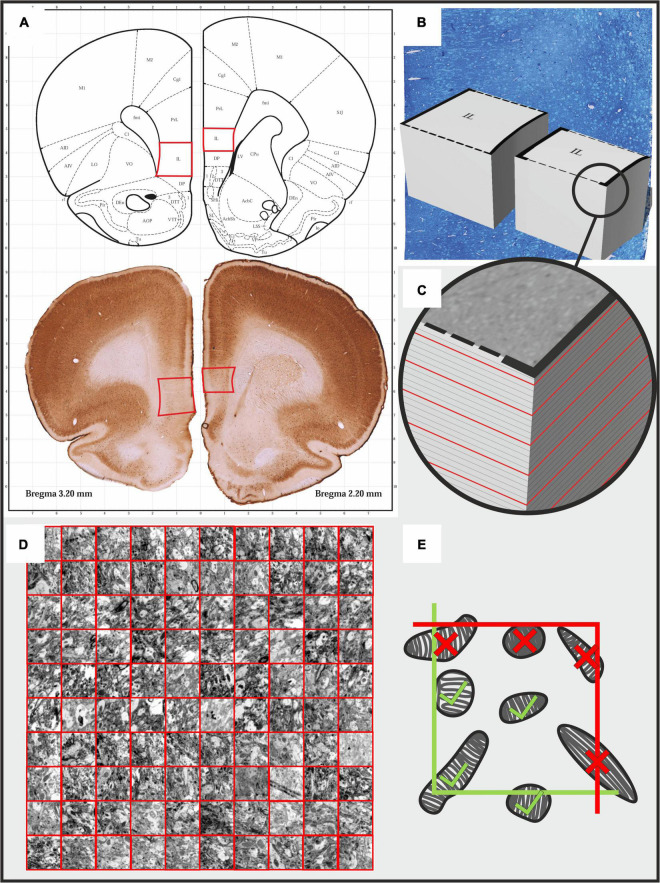
Illustrating the protocol for the systematic quantitative analysis of mitochondrial morphology and number in the infralimbic cortex of rats. **(A)** Two pieces of 80 μm thick coronal sections from the prefrontal cortex were selected between Bregma coordinates of 2.2 - 2.3 mm. Afterward, tissue fragments containing the infralimbic cortices were cut out and re-embedded in durcupan resin to produce tissue blocks for further ultra-sectioning. **(B,C)** First, semi-thin sections were cut and stained with toluidine blue dye to determine the exact area for further ultra-sectioning. **(C)** Afterward, serial 60 nm ultrathin sections were sectioned and we collected every 5th sections on single slot copper grids. The red lines on the image indicate the ultrathin sections. From each animal, we examined 10 ultrathin sections with the electron microscope. **(D)** In every ultrathin section, in each cortical layer, we made at least 10 non-overlapping photomicrographs using a sampling line with a random starting point. We analyzed 90 photomicrographs in each cortical layer of each animal. Mitochondria were counted and their cross-sectional profiles were outlined in these 40000× magnified images. **(E)** For the quantification we used an unbiased counting frame (3.87 × 3.87 μm). Mitochondria profiles touching the exclusion (red) lines were not counted.

Brain tissue fixation and preparation for the electron microscopic analysis was carried out according to our standard protocol (Csabai et al., 2017, 2018). Animals were deeply anesthetized with an overdose of sodium pentobarbital (200 mg/ml dissolved in 10% ethanol) and then, transcardially perfused with ice cold 0.9% physiological saline followed by 4% paraformaldehyde containing 0.2% glutaraldehyde in 0.1 M phosphate buffer (pH 7.4). Brains were extracted from the skulls and serial, 80 μm thick, coronal sections were cut using a Vibratome (Leica VT1200 S) throughout the entire prefrontal cortex (between Bregma levels of 4.70 to 2.20) (Paxinos and Watson, 1998). Two sections that included the IL cortex were selected from these serial sections (Figure 1A) and osmicated (1% OsO4 in PB for 60 min at 4°C) and then, dehydrated in graded ethanol where the 70% ethanol contained 1% uranyl acetate. After complete dehydration in ascending ethanol series, the sections were immersed in propylene-oxide and then, into a mixture of propylene-oxide and Durcupan resin. Finally, they were flat-embedded in Durcupan resin (Fluka-Sigma-Aldrich, Hungary). After polymerization at 56°C for 48 h, the sections were viewed under a light microscope, and areas of interest were chosen for re-embedding and electron microscopic sectioning. To select the appropriate region of the IL cortex for ultrathin sectioning, semithin (500 nm) sections were stained with toluidine blue (Figure 1B). The ultrathin (60 nm) sections were cut with a Leica Ultracut UCT microtome and collected on Formvar-coated single slot copper grids, stained with uranyl-acetate and lead citrate (Figure 1C).

**FIGURE 2:**
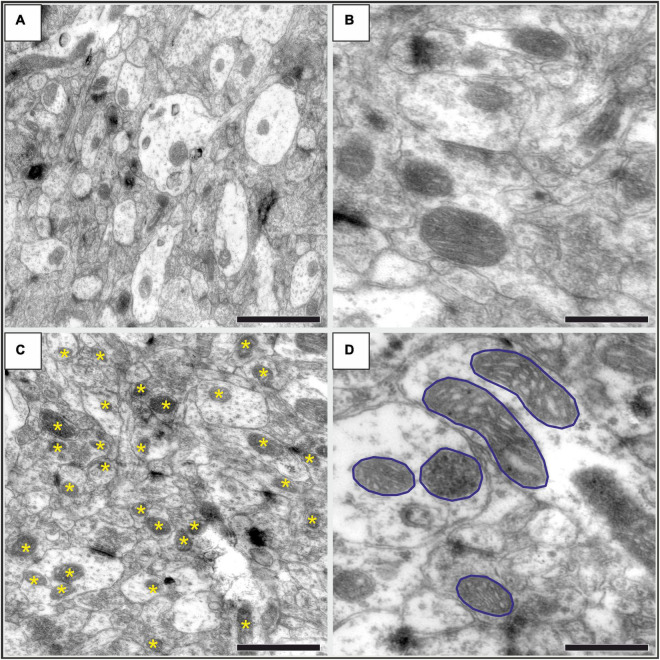
Representative high-resolution electron microscopic images showing the neuropil in the infralimbic cortex in control **(A,B)** and stressed **(C,D)** rats. **(A)** An image with 30000 × magnification, where numerous mitochondria are visible both in dendritic (brighter areas) and in axonal (darker areas) structures. Scale bar: 1 μm. **(A)** Representative images with mitochondria with higher magnification (40000×). The outer and inner mitochondrial membranes, matrix and cristae are clearly visible. Scale bar: 500 nm. **(C)** An image with 30 000× magnification displaying the neuropil in a stressed rat. Asterisks identify the mitochondria as it was marked with the Neurolucida software when we counted them. Scale bar: 1 μm. **(D)** An image with 40000 × magnification showing several mitochondria with their cross-sectional profiles outlined for the morphological analysis. Scale bar: 500 nm.

The ultrathin sections were inspected and microphotographs were taken for the further quantitative analysis via transmission electron microscope (JEOL 1200 EX-II). From every cortical layers, 100-120 high resolution (40000 ×) image series were taken from a random systematic start point along a transect line (Figure 1D). The systematic quantitative analysis was done with the Neurolucida system (Version 7, MicroBrightField, Williston, VT, United States), using unbiased counting frames covering 14,977 μm^2^ of the neuropil tissue (Figure 1E). Two investigators (DC and AST) carried out the quantification in a blinded manner. Mitochondria were counted and their cross-sectional areas were outlined in every 5th images (Figures 2C,D). The first image was identified with a random generator. Quantitative data (mitochondria numbers and contour details) were exported to Microsoft Excel 365 where data was grouped for statistical analysis.

We also determined the volume of the IL cortex based on the Cavalieri’s principle. The volume measurements were done as follows: we collected a series of 80 μm thick coronal sections covering the entire IL cortex starting from 3.5 mm to 2.0 mm relative to Bregma (Paxinos and Watson, 1998) from every animal. Every 3rd serial section (5-6 sections/animal) were stained with Cresyl Violet and thereafter analyzed with a Nikon Eclipse Ti-U bright field microscope, using a 4 × objective. We outlined the borders of the IL cortex using the Neurolucida software based on the descriptions of the stereotaxic rat brain atlas of Paxinos and Watson (1998). In these Nissl stained sections, we also measured the cross-sectional areas of cortical layers I, II, III, V, VI of the infralimbic cortex (note that the IL has no layer IV). The boundaries between the cortical layers were recognized according to the description given by Gabbott et al. (1997). The volume of the IL cortex was calculated by multiplying the cross-sectional areas with the thickness of the sections and by the intersection interval.

Results are presented here as the mean ± SEM. GraphPad Prism 7.0 package was used for the statistical analysis. We used the Shapiro-Wilk normality test to verify that the data had Gaussian distribution, but we did not specifically assess the homogeneity of variances. Results of the sucrose preference test were analyzed with one-way ANOVA, followed by Dunnets’s Multiple Comparison Test. Group values of mitochondria numbers and morphology were compared with two-tailed unpaired Student *t*-test. Mitochondria numbers and densities in the different cortical layers were compared with 2-way ANOVA (stress × cortical layer) followed by Sidak’s multiple comparisons *post hoc* test. Results were considered significant when *P* value was < 0.05.

## Results

**FIGURE 3:**
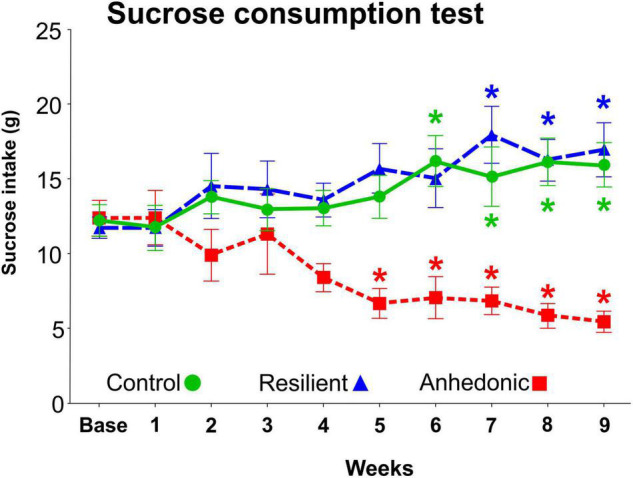
Results of the sucrose consumption test. The sucrose intake of the rats was determined every week in order to behaviorally phenotype the animals and to evaluate their response to the chronic mild stress exposure. The original CMS study involved 335 rats. 260 rats were subjected to the CMS protocol, whereas the remaining 75 rats were kept undisturbed in separate rooms and used as controls. About 40% of the stressed rats (*n* = 98) were vulnerable to stress and significantly reduced their sucrose intake by > 30%. These animals were then labeled as “anhedonic” rats. Out of these 98 anhedonic rats 4 animals were selected for the present quantitative electron microscopic analysis based on their marked reduction in sucrose intake. About 20% of the stressed rats (*n* = 46) were unaffected by the stress exposure and even increased their sucrose intake, similarly to the control animals. These stress resistant rats were labeled as “stress-resilient”. The remaining stressed animals (*n* = 116) with intermediate sucrose consumption phenotype were excluded from the experiment. Baseline sucrose consumption was defined as the mean sucrose consumption during three sucrose tests conducted before the stress protocol started. Statistics: one-way ANOVA, followed by Dunnets’s multiple comparison tests where results are compared of the baseline sucrose consumption of the corresponding group. *P < 0.05 versus baseline value of the same group.

We used the sucrose consumption test to evaluate the response of the animals to the CMS exposure, since this test provides a reliable behavioral readout of anhedonia (Willner, 1997). From the 260 stressed rats, 98 animals (38%) displayed anhedonic phenotype, i.e., these animals reduced their sucrose intake by more than 30% compared to their baseline intake value (Figure 3). A much smaller fraction of the stressed rats, 46 animals (18%) showed stress-resilient phenotype, i.e., these animals did not decrease their sucrose intake during the stress procedures. The remaining 116 stressed rats had an intermediate phenotype therefore, these animals were excluded from the experiment. From the 98 anhedonic animals, we selected 4 rats which had pronounced reduction in sucrose consumption and the brains of these animals were processed for the electron microscopic analysis.

Examples of representative electron micrographs from the present study are shown on Figure 2. We analyzed 2,152 electron microscopic images, and 36,712 mitochondria were counted and examined in the four control rats. Similarly, 2,101 electron microscopic images were examined and 30,279 mitochondria were counted in the infralimbic cortex of the four stressed animals. On average, we analyzed 90 electron microscopic images in each cortical layer in each animal. We not only quantified the mitochondria, but we also delineated the cross-sectional areas of the mitochondria (for every 10th examined mitochondria) and afterward with the aid of the Neurolucida Explorer software we determined the following morphological parameters: ferret; aspect ratio; compactness; form factor and convexity. However, this analysis of mitochondrial morphology did not yield any statistically significant difference between the control and stressed animals. Results of the morphological evaluation are summarized in Table 1.

**TABLE 1:** Comparisons of mitochondrial morphology.

	Control	Stress	Student *t* test	
Perimeter (nm)	929.70 ± 31.17	977.90 ± 31.49	*t* = 1.087	*P* = 0.319
Area (μm^2^)	0.06 ± 0.01	0.07 ± 0.01	*t* = 1.046	*P* = 0.336
Feret Min	186.30 ± 8.21	203.80 ± 17.90	*t* = 0.888	*P* = 0.409
Feret Max	352.80 ± 10.03	365.00 ± 15.39	*t* = 0.665	*P* = 0.531
Aspect Ratio	1.89 ± 0.02	1.83 ± 0.07	*t* = 0.710	*P* = 0.504
Compactness	0.75 ± 0.00	0.76 ± 0.01	*t* = 0.941	*P* = 0.383
Form Factor	0.83 ± 0.00	0.85 ± 0.01	*t* = 0.894	*P* = 0.406
Convexity	0.998	0.999	*t* = 1.792	*P* = 1.123

*We examined mitochondrial morphology the following way: The cross-sectional boundaries of the mitochondria were outlined on the electron microscopic images using the Neurolucida software. Afterwards another software (NeuroExplorer) scrutinized these cross-sectional outlines and quantified the following features: 1) Perimeter is the length of the contour. 2) Area is the 2-dimensional cross-sectional area enclosed within the boundary of a closed contour. 3) Feret Min and Feret Max are the smallest and largest dimensions of the contour. 4) Aspect ratio is the ratio of maximal and minimal diameters. This indicates the flatness of the shapes. 5) Compactness describes the relationship between the area and the maximum diameter. 6) Form Factor indicates the smoothness of the perimeter. The higher this value is, the smoother the perimeter of the shape is. 7) Convexity indicates the profile complexity – convex objects have a convexity value of 1. Data are expressed as mean ± S.E.M.*

**FIGURE 4:**
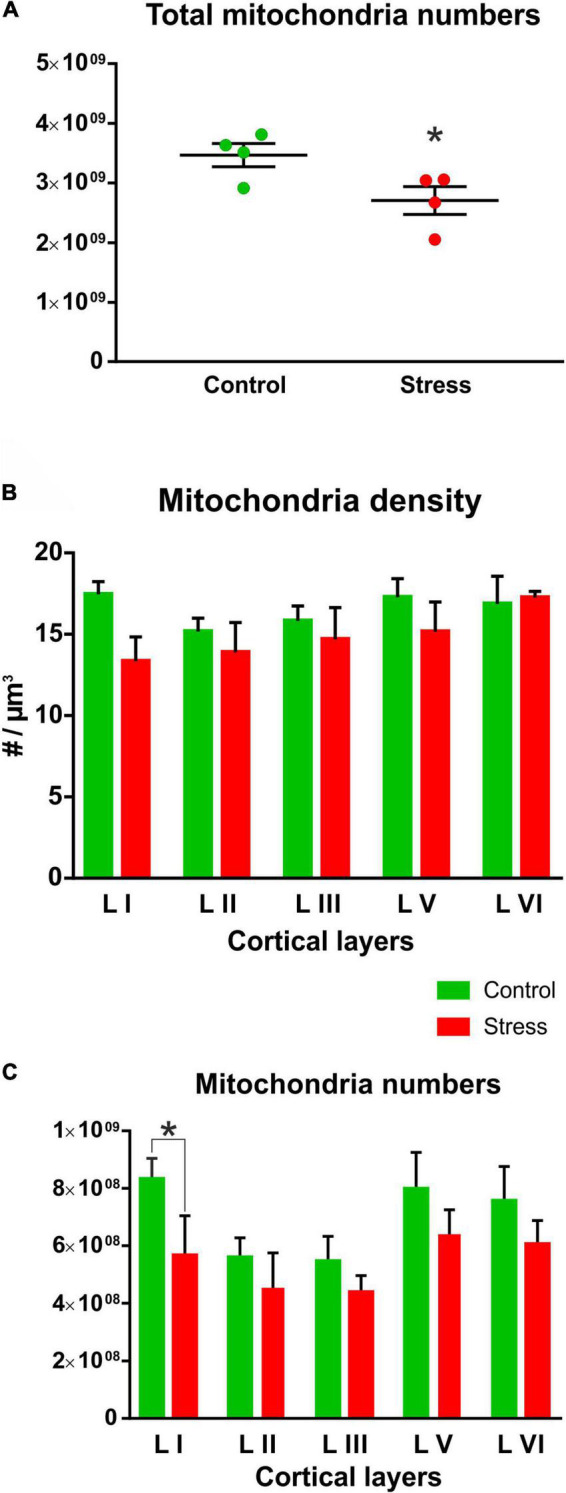
**(A)** Chronic mild stress significantly reduced the total number of mitochondria in the infralimbic cortex (*t* test, *P = 0.04). Total mitochondria numbers in the infralimbic cortex were calculated by multiplying mitochondria densities with the volume of the infralimbic cortex. **(B)** Mitochondria densities were only marginally reduced in the different cortical layers of the stressed animals, but these changes were not statistically significant (stress effect: *P* = 0.07). **(C)** Mitochondria numbers in the different cortical layers were compared with two-way ANOVA (stress × cortical layer) which revealed a significant difference between cortical layers (*P* < 0.0001) and also a significant stress effect (*P* < 0.0001). Sidak’s *post hoc* test found significant difference between control and stressed animals only in the first cortical layer (*P = 0.004). Mitochondria numbers within the cortical layers were calculated by multiplying mitochondria densities with the different cortical layer volumes.

The total number of mitochondria had normal Gaussian distribution both in the Control and Stress animals. Shapiro-Wilk normality test found no significant difference in the Control (*W* = 0.893, *P* = 0.399) and Stress (*W* = 0.847, *P* = 0.218) groups therefore, these values passed the normality test in both groups. When comparing the total number of mitochondria between the Control and Stress groups, we found that the stressed rats had significantly reduced number of mitochondria in their infralimbic cortex (*t* = 2.498, *P* = 0.046; Figure 4A).

Mitochondrial density values in the cortical layers had normal distribution both in the Control and Stress groups. Shapiro-Wilk normality test found no significant difference in the Control (*W* = 0.899, *P* = 0.407) and Stress (*W* = 0.930, *P* = 0.596) groups therefore, the values passed the normality test in both groups. When we compared the mitochondrial density values between the two groups we found not statistical difference, only trends for reduction (Figure 4B).

We also calculated the total number of mitochondria in the different cortical layers. These numbers were calculated from cortical layer volumes and mitochondrial density. Total mitochondria numbers in the different cortical layers also showed normal distribution both in the Control and Stress groups. Shapiro-Wilk normality test found no significant difference in the Control (*W* = 0.33, *P* = 0.147) and Stress (*W* = 0.887, *P* = 0.345) groups therefore, the values passed the normality test in both groups. Comparison of mitochondria numbers in the different cortical layers revealed a significantly reduced number of mitochondria in layer I (Figure 4C). Two-way ANOVA (stress × cortical layer) revealed a highly significant difference between cortical layers [F (4, 30) = 40.23, *P* < 0.0001] and between the two groups [F (1, 30) = 25.59, *P* < 0.0001)]. Sidak’s *post hoc* analysis found significant difference between control and stressed animals in cortical layer I (*t* = 3.76, *P* = 0.004). In the other layers, we found a clear trend of stress-induced reduction, but statistical difference emerged only when we combined data from the five layers as it is shown on Figure 4A.

## Discussion

This is the first systematic electron microscopic investigation examining the effect of chronic stress on mitochondria numbers and morphology in the medial PFC of rats. Our present preliminary findings extend our earlier report where we found decreased number of synapses in the infralimbic cortex of the same stressed animals (Csabai et al., 2018). Now, we report that the total number of mitochondria was reduced in the infralimbic cortex of the stressed animals. This finding is a collective result of the diminished mitochondrial density in the different cortical layers together with the stress-induced volume shrinkage that we have observed in the infralimbic cortex of these animals (for details see Figure 5 of Csabai et al., 2018). However, we could not detect any stress-induced alteration in the cross-sectional mitochondrial morphology. Notably, our present preliminary data does not provide any specific information on the potential functional consequences of this mitochondrial loss.

One potential explanation for the present finding is that neurons die and this cell death contributes to the volume shrinkage and to the reduction in mitochondria numbers. For example, a study investigating the consequences of a single-prolonged stress exposure in an animal model of PTSD reported on stress-induced increase of apoptotic cells in the hippocampus, together with release of cytochrome c from the mitochondria into the cytosol and with morphological abnormalities of the mitochondria (i.e., vacuolar degeneration, cristae fragmentation, exterior membrane disruption, and swollen mitochondria) (Li et al., 2010). It has also been shown that mitochondrial protein pathways seem to mediate the effect of stress on neurodegeneration (Lopes et al., 2017). Another possibility is that stress alters neuronal complexity, the dendritic arbors shrink, which leads to the significant loss of neuropil. Numerous studies have reported on stress-induced reduction of dendritic complexity and axon loss in the infralimbic cortex of rats (Izquierdo et al., 2006; Perez-Cruz et al., 2009; Csabai et al., 2018; Wellman et al., 2020). In the present study, the most pronounced reduction of mitochondria numbers was detected in layer I of the IL cortex. This layer is the molecular layer, and consists mainly the most distal part of the apical dendritic tufts of pyramidal neurons and horizontally oriented axons. This layer contains mainly glial cells and only a few scattered GABAergic neurons are found here. The explanation for this pronounced change in mitochondrial numbers in layer I could be the stress-induced shrinkage of the apical dendritic tree and loss of axons (Perez-Cruz et al., 2009; Csabai et al., 2018; Wellman et al., 2020).

Mitochondria play a key role in chemical energy production for the cells and execute a wide range of additional functions including the production of reactive oxygen species, buffering intracellular calcium and regulating neuronal structure and function. Importantly, glucocorticoid hormones have a direct influence on mitochondrial DNA transcription and mitochondrial function (Du et al., 2009; Hunter et al., 2016). Mitochondrial glucocorticoid receptors seem to mediate the glucocorticoid-induced apoptosis (Sionov et al., 2006). It has been proposed that individuals with inadequate mitochondrial function could be vulnerable to the stress-induced depletion of the brain’s energy resources and, by that, to the development of psychopathologies (Morava and Kozicz, 2013; Filiou and Sandi, 2019; Daniels et al., 2020). Therefore, dysfunctional mitochondria have been pointed out as a central feature of neuropsychiatric disorders (Bansal and Kuhad, 2016; Pei and Wallace, 2018; Filiou and Sandi, 2019; Allen et al., 2021).

In the present study, the rats exposed to chronic mild stress were characterized based on their anhedonic behavior, i.e., the amount of sucrose solution they consumed during the sucrose consumption test. Anhedonia, or reward sensitivity, is typically assessed with the use of sucrose consumption or preference. The anhedonic behavior, in response to CMS, can be detected both with the single-bottle consumption tests and with the two-bottle, sucrose-water, preference tests (Willner, 1997). Furthermore, it has been shown that the observed decrease in sucrose consumption is specific and does not reflect a general decrease in fluid consumption (e.g., decreased thirst), since intake of pure water is unaffected by the CMS paradigm (Willner et al., 1992; Henningsen et al., 2012). Therefore, the decreased sucrose consumption is not a physiological but a mental phenomenon. The reduced sucrose consumption implies a diminished reward sensitivity. This has been confirmed by assessing the rewarding properties of food, which are also reduced, as indicated by an attenuation of food-induced place preference conditioning (Papp et al., 1991; Muscat and Willner, 1992).

In the present study, we did not assess any other behavioral changes in response to chronic stress. However, our research group has been working with this animal model for many years and we have demonstrated several behavioral alterations in the chronically stressed animals. For example, rats exposed to the same paradigm develop disturbed circadian rhythms (Christiansen et al., 2012, 2016) and learning deficits in cognitive tasks which assess fronto-cortical cognitive performances (Czéh et al., 2018; Martis et al., 2018). In addition to that, we have described reduced dentate cytogenesis (Jayatissa et al., 2006), and reduced number of granule cells in the hippocampus (Jayatissa et al., 2008) as well as changes in GABAergic neuronal cell numbers in the hippocampus and in the frontal cortex (Czéh et al., 2015, 2018; Varga et al., 2017). Using magnetic resonance imaging we could also detect microstructural and metabolic brain alterations (Delgado y Palacios et al., 2011; Khan et al., 2016, 2018). Overall, this model is one of the best characterized animal model for depression (Willner, 2005, 2016).

A major limitation of the present study is that the data have been generated from a small number of animals. We should point out however, that quantitative electron microscopic studies often involve only a few animals (see e.g., Hajszan et al., 2009, 2010; Kikuchi et al., 2020; Santuy et al., 2020; Huzian et al., 2021). The main reason for this is that a systematic quantitative electron microscopic analysis is a time-consuming and labor-intensive method. On the other hand, the quantified values, e.g., synapse counts in a specific brain structure, appear to have a low variation, i.e., their standard deviations are in the range of 5-10% of the mean (Hajszan et al., 2009, 2010). Another limitation of the present study is that we did not include any stress-resilient rats in our histopathological analysis. We should emphasize that this study was a preliminary investigation. First, we wanted to see whether stress has any effect on mitochondria numbers and morphology therefore, we selected animals which were most susceptible to the stress exposure. According to our previous studies, the stress-resilient animals often displayed cellular changes with values in-between the control and anhedonic animals (Czéh et al., 2015, 2018). Future, more detailed studies using larger number of animals should confirm our present findings. Including stress-resilient animals in the investigations should also answer the question whether the different behavioral phenotypes correlate with the mitochondrial loss.

In summary, the present data provide further experimental evidence that stress can affect neuronal mitochondria. Our observations however, provide no information on the functional consequences. Therefore, further studies are necessary to confirm the concept that the stress-induced mitochondrial damage can sensitize neurons to additional injuries leading to impaired neuronal functioning and to the development of mental disorders.

## Data Availability Statement

The original contributions presented in the study are included in the article/supplementary material, further inquiries can be directed to the corresponding author/s.

## Ethics Statement

The animal study was reviewed and approved by Danish National Committee for Ethics in Animal Experimentation.

## Author Contributions

DC: methodology, investigation, formal analysis, visualization, and writing - original draft. AS-T: investigation and formal analysis. OW: funding acquisition, resources, and writing - review & editing. BC: conceptualization, resources, writing – original draft, review, and editing, project administration, funding acquisition, and supervision. All authors have read and agreed to the published version of the manuscript.

## Conflict of Interest

The authors declare that the research was conducted in the absence of any commercial or financial relationships that could be construed as a potential conflict of interest.

## Publisher’s Note

All claims expressed in this article are solely those of the authors and do not necessarily represent those of their affiliated organizations, or those of the publisher, the editors and the reviewers. Any product that may be evaluated in this article, or claim that may be made by its manufacturer, is not guaranteed or endorsed by the publisher.

## Abbreviations

CMS: chronic mild stress
IL: infralimbic cortex
mPFC: medial prefrontal cortex
PFC: prefrontal cortex
PTSD: Post-traumatic stress disorder.

## References

[B1] Allen J. Caruncho H. J. Kalynchuk L. E. (2021). Severe life stress, mitochondrial dysfunction, and depressive behavior: A pathophysiological and therapeutic perspective. *Mitochondrion* 56 111–117. 10.1016/j.mito.2020.11.010 33220501

[B2] Arnsten A. F. (2015). Stress weakens prefrontal networks: molecular insults to higher cognition. *Nat. Neurosci.* 18 1376–1385. 10.1038/nn.4087 26404712 PMC4816215

[B3] Bansal Y. Kuhad A. (2016). Mitochondrial Dysfunction in Depression. *Curr. Neuropharmacol.* 14 610–618. 10.2174/1570159x14666160229114755 26923778 PMC4981740

[B4] Choi G. E. Han H. J. (2021). Glucocorticoid impairs mitochondrial quality control in neurons. *Neurobiol. Dis.* 152:105301. 10.1016/j.nbd.2021.105301 33609641

[B5] Christiansen S. Bouzinova E. V. Palme R. Wiborg O. (2012). Circadian activity of the hypothalamic-pituitary-adrenal axis is differentially affected in the rat chronic mild stress model of depression. *Stress* 15 647–657. 10.3109/10253890.2011.654370 22217141

[B6] Christiansen S. L. Højgaard K. Wiborg O. Bouzinova E. V. (2016). Disturbed diurnal rhythm of three classical phase markers in the chronic mild stress rat model of depression. *Neurosci. Res.* 110 43–48. 10.1016/j.neures.2016.03.002 27033803

[B7] Csabai D. Seress L. Varga Z. Ábrahám H. Miseta A. Wiborg O. (2017). Electron Microscopic Analysis of Hippocampal Axo-Somatic Synapses in a Chronic Stress Model for Depression. *Hippocampus* 27 17–27. 10.1002/hipo.22650 27571571 PMC5215622

[B8] Csabai D. Wiborg O. Czéh B. (2018). Reduced Synapse and Axon Numbers in the Prefrontal Cortex of Rats Subjected to a Chronic Stress Model for Depression. *Front. Cell Neurosci.* 12:24. 10.3389/fncel.2018.00024 PMC5797661 29440995

[B9] Czéh B. Vardya I. Varga Z. Febbraro F. Csabai D. Martis L. S. (2018). Long-Term Stress Disrupts the Structural and Functional Integrity of GABAergic Neuronal Networks in the Medial Prefrontal Cortex of Rats. *Front. Cell Neurosci.* 12:148. 10.3389/fncel.2018.00148 29973870 PMC6020798

[B10] Czéh B. Varga Z. K. Henningsen K. Kovács G. L. Miseta A. Wiborg O. (2015). Chronic stress reduces the number of GABAergic interneurons in the adult rat hippocampus, dorsal-ventral and region-specific differences. *Hippocampus* 25 393–405. 10.1002/hipo.22382 25331166

[B11] Daniels T. E. Olsen E. M. Tyrka A. R. (2020). Stress and Psychiatric Disorders: the Role of Mitochondria. *Annu. Rev. Clin. Psychol.* 16 165–186. 10.1146/annurev-clinpsy-082719-104030 32092280 PMC8007172

[B12] Delgado y Palacios R. Campo A. Henningsen K. Verhoye M. Poot D. (2011). Magnetic resonance imaging and spectroscopy reveal differential hippocampal changes in anhedonic and resilient subtypes of the chronic mild stress rat model. *Biol. Psychiatry* 70 449–457. 10.1016/j.biopsych.2011.05.014 21762877

[B13] Du J. Wang Y. Hunter R. Wei Y. Blumenthal R. Falke C. (2009). Dynamic regulation of mitochondrial function by glucocorticoids. *Proc. Natl. Acad. Sci. U.S.A.* 106 3543–3548. 10.1073/pnas.0812671106 19202080 PMC2637276

[B14] Filiou M. D. Sandi C. (2019). Anxiety and Brain Mitochondria: A Bidirectional Crosstalk. *Trends Neurosci.* 42 573–588. 10.1016/j.tins.2019.07.002 31362874

[B15] Flak J. N. Solomon M. B. Jankord R. Krause E. G. Herman J. P. (2012). Identification of chronic stress-activated regions reveals a potential recruited circuit in rat brain. *Eur. J. Neurosci.* 36 2547–2555. 10.1111/j.1460-9568.2012.08161.x 22789020 PMC4538599

[B16] Gabbott P. L. Dickie B. G. Vaid R. R. Headlam A. J. Bacon S. J. (1997). Local-circuit neurones in the medial prefrontal cortex (areas 25, 32 and 24b) in the rat: morphology and quantitative distribution. *J. Comp. Neurol.* 377 465–499. 9007187 10.1002/(sici)1096-9861(19970127)377:4<465::aid-cne1>3.0.co;2-0

[B17] Gorka Z. Moryl E. Papp M. (1996). Effect of chronic mild stress on circadian rhythms in the locomotor activity in rats. *Pharmacol. Biochem. Behav.* 54 229–234. 10.1016/0091-3057(95)02173-6 8728562

[B18] Hajszan T. Dow A. Warner-Schmidt J. L. Szigeti-Buck K. Sallam N. L. Parducz A. (2009). Remodeling of hippocampal spine synapses in the rat learned helplessness model of depression. *Biol. Psychiatry.* 65 392–400. 10.1016/j.biopsych.2008.09.031 19006787 PMC2663388

[B19] Hajszan T. Szigeti-Buck K. Sallam N. L. Bober J. Parducz A. Maclusky N. J. (2010). Effects of estradiol on learned helplessness and associated remodeling of hippocampal spine synapses in female rats. *Biol. Psychiatry.* 67 168–174. 10.1016/j.biopsych.2009.08.017 19811775 PMC2794927

[B20] Henningsen K. Andreasen J. T. Bouzinova E. V. Jayatissa M. N. Jensen M. S. Redrobe J. P. (2009). Cognitive deficits in the rat chronic mild stress model for depression: relation to anhedonic-like responses. *Behav. Brain Res.* 198 136–141. 10.1016/j.bbr.2008.10.039 19038290

[B21] Henningsen K. Palmfeldt J. Christiansen S. Baiges I. Bak S. Jensen O. N. (2012). Candidate hippocampal biomarkers of susceptibility and resilience to stress in a rat model of depression. *Mol. Cell Proteom.* 11:M111.016428. 10.1074/mcp.M111.016428 22311638 PMC3394954

[B22] Hinwood M. Tynan R. J. Day T. A. Walker F. R. (2011). Repeated social defeat selectively increases δFosB expression and histone H3 acetylation in the infralimbic medial prefrontal cortex. *Cereb. Cortex* 21 262–271. 10.1093/cercor/bhq080 20513656

[B23] Holmes A. Wellman C. L. (2009). Stress-induced prefrontal reorganization and executive dysfunction in rodents. *Neurosci. Biobehav. Rev.* 33 773–783. 10.1016/j.neubiorev.2008.11.005 19111570 PMC2941982

[B24] Hunter R. G. Seligsohn M. Rubin T. G. Griffiths B. B. Ozdemir Y. Pfaff D. W. (2016). Stress and corticosteroids regulate rat hippocampal mitochondrial DNA gene expression via the glucocorticoid receptor. *Proc. Natl. Acad. Sci. U.S.A.* 113 9099–9104. 10.1073/pnas.1602185113 27457949 PMC4987818

[B25] Huzian O. Baka J. Csakvari E. Dobos N. Leranth C. Siklos L. (2021). Stress Resilience is Associated with Hippocampal Synaptoprotection in the Female Rat Learned Helplessness Paradigm. *Neuroscience* 459 85–103. 10.1016/j.neuroscience.2021.01.029 33524494

[B26] Izquierdo A. Wellman C. L. Holmes A. (2006). Brief uncontrollable stress causes dendritic retraction in infralimbic cortex and resistance to fear extinction in mice. *J. Neurosci.* 26 5733–5738. 10.1523/JNEUROSCI.0474-06.2006 16723530 PMC6675270

[B27] Jayatissa M. N. Bisgaard C. Tingström A. Papp M. Wiborg O. (2006). Hippocampal cytogenesis correlates to escitalopram-mediated recovery in a chronic mild stress rat model of depression. *Neuropsychopharmacology* 31 2395–2404. 10.1038/sj.npp.1301041 16482085

[B28] Jayatissa M. N. Bisgaard C. F. West M. J. Wiborg O. (2008). The number of granule cells in rat hippocampus is reduced after chronic mild stress and re-established after chronic escitalopram treatment. *Neuropharmacology* 54 530–541. 10.1016/j.neuropharm.2007.11.009 18164735

[B29] Khan A. R. Chuhutin A. Wiborg O. Kroenke C. D. Nyengaard J. R. Hansen B. (2016). Biophysical modeling of high field diffusion MRI demonstrates micro-structural aberration in chronic mild stress rat brain. *Neuroimage* 142 421–430. 10.1016/j.neuroimage.2016.07.001 27389790 PMC5159333

[B30] Khan A. R. Geiger L. Wiborg O. Czéh B. (2020). Stress-Induced Morphological, Cellular and Molecular Changes in the Brain-Lessons Learned from the Chronic Mild Stress Model of Depression. *Cells* 9:1026. 10.3390/cells9041026 32326205 PMC7226496

[B31] Khan A. R. Hansen B. Wiborg O. Kroenke C. D. Jespersen S. N. (2018). Diffusion MRI and MR spectroscopy reveal microstructural and metabolic brain alterations in chronic mild stress exposed rats: A CMS recovery study. *Neuroimage* 167 342–353. 10.1016/j.neuroimage.2017.11.053 29196269 PMC5845761

[B32] Kikuchi T. Gonzalez-Soriano J. Kastanauskaite A. Benavides-Piccione R. Merchan-Perez A. DeFelipe J. (2020). Volume Electron Microscopy Study of the Relationship Between Synapses and Astrocytes in the Developing Rat Somatosensory Cortex. *Cereb. Cortex* 30 3800–3819. 10.1093/cercor/bhz343 31989178 PMC7233003

[B33] Li X. M. Han F. Liu D. J. Shi Y. X. (2010). Single-prolonged stress induced mitochondrial-dependent apoptosis in hippocampus in the rat model of post-traumatic stress disorder. *J. Chem. Neuroanat.* 40 248–255. 10.1016/j.jchemneu.2010.07.001 20624456

[B34] Lopes S. Teplytska L. Vaz-Silva J. Dioli C. Trindade R. Morais M. (2017). Tau Deletion Prevents Stress-Induced Dendritic Atrophy in Prefrontal Cortex: role of Synaptic Mitochondria. *Cereb. Cortex* 27 2580–2591. 10.1093/cercor/bhw057 27073221

[B35] Lucassen P. J. Pruessner J. Sousa N. Almeida O. F. Van Dam A. M. Rajkowska G. (2014). Neuropathology of stress. *Acta Neuropathol.* 127 109–135. 10.1007/s00401-013-1223-5 24318124 PMC3889685

[B36] Martis L. S. Brision C. Holmes M. C. Wiborg O. (2018). Resilient and depressive-like rats show distinct cognitive impairments in the touchscreen paired-associates learning (PAL) task. *Neurobiol. Learn. Mem.* 155 287–296. 10.1016/j.nlm.2018.08.014 30138691

[B37] McEwen B. S. Bowles N. P. Gray J. D. Hill M. N. Hunter R. G. Karatsoreos I. N. (2015). Mechanisms of stress in the brain. *Nat. Neurosci.* 18 1353–1363. 10.1038/nn.4086 26404710 PMC4933289

[B38] Moench K. M. Maroun M. Kavushansky A. Wellman C. (2015). Alterations in neuronal morphology in infralimbic cortex predict resistance to fear extinction following acute stress. *Neurobiol. Stress* 3 23–33. 10.1016/j.ynstr.2015.12.002 26844245 PMC4730795

[B39] Morava E. Kozicz T. (2013). Mitochondria and the economy of stress (mal)adaptation. *Neurosci. Biobehav. Rev.* 37 668–680. 10.1016/j.neubiorev.2013.02.005 23415702

[B40] Muscat R. Willner P. (1992). Suppression of sucrose drinking by chronic mild unpredictable stress: a methodological analysis. *Neurosci. Biobehav. Rev.* 16 507–517. 10.1016/s0149-7634(05)80192-7 1480347

[B41] Papp M. Willner P. Muscat R. (1991). An animal model of anhedonia: attenuation of sucrose consumption and place preference conditioning by chronic unpredictable mild stress. *Psychopharmacology* 104 255–259. 10.1007/BF02244188 1876670

[B42] Paxinos G. Watson C. (1998). *The Rat Brain in Stereotaxic Coordinates.* Cambridge: Academic Press.

[B43] Pei L. Wallace D. C. (2018). Mitochondrial Etiology of Neuropsychiatric Disorders. *Biol. Psychiatry* 83 722–730. 10.1016/j.biopsych.2017.11.018 29290371 PMC5891364

[B44] Perez-Cruz C. Simon M. Flügge G. Fuchs E. Czéh B. (2009). Diurnal rhythm and stress regulate dendritic architecture and spine density of pyramidal neurons in the rat infralimbic cortex. *Behav. Brain Res.* 205 406–413. 10.1016/j.bbr.2009.07.021 19643147

[B45] Picard M. Juster R. P. McEwen B. S. (2014). Mitochondrial allostatic load puts the ‘gluc’ back in glucocorticoids. *Nat. Rev. Endocrinol.* 10 303–310. 10.1038/nrendo.2014.22 24663223

[B46] Picard M. McEwen B. S. Epel E. S. Sandi C. (2018). An energetic view of stress: focus on mitochondria. *Front. Neuroendocrinol.* 49:72–85. 10.1016/j.yfrne.2018.01.001 29339091 PMC5964020

[B47] Santuy A. Tomás-Roca L. Rodríguez J. R. González-Soriano J. Zhu F. Qiu Z. (2020). Estimation of the number of synapses in the hippocampus and brain-wide by volume electron microscopy and genetic labeling. *Sci. Rep.* 10:14014. 10.1038/s41598-020-70859-5 32814795 PMC7438319

[B48] Shutt T. E. McBride H. M. (2013). Staying cool in difficult times: mitochondrial dynamics, quality control and the stress response. *Biochim. Biophys. Acta* 1833 417–424. 10.1016/j.bbamcr.2012.05.024 22683990

[B49] Sionov R. V. Cohen O. Kfir S. Zilberman Y. Yefenof E. (2006). Role of mitochondrial glucocorticoid receptor in glucocorticoid-induced apoptosis. *J. Exp. Med.* 203 189–201. 10.1084/jem.20050433 16390935 PMC2118093

[B50] Varga Z. Csabai D. Miseta A. Wiborg O. Czéh B. (2017). Chronic stress affects the number of GABAergic neurons in the orbitofrontal cortex of rats. *Behav. Brain Res.* 316 104–114. 10.1016/j.bbr.2016.08.030 27555539

[B51] Weger M. Alpern D. Cherix A. Ghosal S. Grosse J. Russeil J. (2020). Mitochondrial gene signature in the prefrontal cortex for differential susceptibility to chronic stress. *Sci. Rep.* 10:18308. 10.1038/s41598-020-75326-9 33110158 PMC7591539

[B52] Wellman C. L. Bollinger J. L. Moench K. M. (2020). Effects of stress on the structure and function of the medial prefrontal cortex: insights from animal models. *Int. Rev. Neurobiol.* 150 129–153. 10.1016/bs.irn.2019.11.007 32204829 PMC9483990

[B53] Wiborg O. (2013). Chronic mild stress for modeling anhedonia. *Cell Tissue Res.* 354 155–169. 10.1007/s00441-013-1664-0 23801433

[B54] Willner P. (1997). Validity, reliability and utility of the chronic mild stress model of depression: a 10-year review and evaluation. *Psychopharmacology* 134 319–329. 10.1007/s002130050456 9452163

[B55] Willner P. (2005). Chronic mild stress (CMS) revisited: consistency and behavioural-neurobiological concordance in the effects of CMS. *Neuropsychobiology* 52 90–110. 10.1159/000087097 16037678

[B56] Willner P. (2016). The chronic mild stress (CMS) model of depression: history, evaluation and usage. *Neurobiol. Stress* 6 78–93. 10.1016/j.ynstr.2016.08.002 28229111 PMC5314424

[B57] Willner P. Muscat R. Papp M. (1992). Chronic mild stress-induced anhedonia: a realistic animal model of depression. *Neurosci. Biobehav. Rev.* 16 525–534. 10.1016/s0149-7634(05)80194-0 1480349

[B58] Woo E. Sansing L. H. Arnsten A. F. T. Datta D. (2021). Chronic Stress Weakens Connectivity in the Prefrontal Cortex: architectural and Molecular Changes. *Chronic Stress* 5:24705470211029254. 10.1177/24705470211029254 34485797 PMC8408896

